# Serotonergic regulation of neuronal excitability and glutamatergic synaptic transmission in rat claustrum

**DOI:** 10.3389/fncel.2026.1951375

**Published:** 2026-10-13

**Authors:** Renee Rouleau, Saida Oubraim, Lizemarie Cirone, Roh-Yu Shen, Samir Haj-Dahmane

**Affiliations:** 1 Department of Pharmacology and Toxicology, Jacobs School of Medicine and Biomedical Sciences, University at Buffalo, State University of New York, Buffalo, NY, United States; 2 University at Buffalo Neuroscience Program, Jacobs School of Medicine and Biomedical Sciences, University at Buffalo, State University of New York, Buffalo, NY, United States; 3 Department of Neurobiology and Behavior, Cornell University, Ithaca, NY, United States

**Keywords:** 5-HT2AR, claustrum, excitability, psychedelic, serotonin

## Abstract

The claustrum (CLA) receives dense serotonergic innervation from dorsal raphe nucleus (DRN) serotonin (5-HT) neurons, which are implicated in regulating sleep and stress homeostasis. However, the mechanisms by which 5-HT regulates the excitability of CLA neurons remain poorly defined. In the present study, we combined whole-cell patch-clamp recordings, 5-HT uncaging, and receptor pharmacology to examine the effects of 5-HT on the excitability of CLA neurons and their glutamatergic synapses. We found that 5-HT consistently induced membrane hyperpolarization or depolarization in regular-spiking type I and type II neurons, whereas it predominantly induced hyperpolarization in strongly adapting CLA neurons. The 5-HT-induced membrane hyperpolarization and depolarization were mediated by 5-HT1A receptors (5-HT1ARs) and 5-HT2A receptors (5-HT2ARs), respectively. Furthermore, we showed that activation of presynaptic 5-HT2ARs inhibits glutamatergic synaptic transmission in the CLA by reducing glutamate release. This effect was not mediated by the canonical phospholipase C (PLC) signaling cascade. Collectively, these findings reveal cell type-specific and multifaceted serotonergic modulation of the intrinsic excitability and synaptic transmission in the claustrum.

## Introduction

The claustrum (CLA), a thin sheet-like structure located between the insular cortex and the striatum, is one of the most densely interconnected regions of the brain. It has been implicated in a wide range of higher-order brain functions, including sleep (Narikiyo et al., 2020; Norimoto et al., 2020), attention (Bayat et al., 2018; Goll et al., 2015; Jackson, 2018), decision-making (Chevée et al., 2022; Smith et al., 2019), and consciousness (Crick and Koch, 2005; Liaw and Augustine, 2023). CLA neurons project to nearly every cortical area (Atlan et al., 2017; Wang et al., 2017) and receive reciprocal inputs from most cortical regions, with the strongest bidirectional connections arising from the prefrontal cortex and anterior cingulate cortex (Torgerson and Van Horn, 2014; Kim et al., 2016; Atlan et al., 2017; Kitanishi and Matsuo, 2017). Through these widespread reciprocal connections, the CLA modulates cortical activity, at least in part, through feed-forward inhibitory circuits in which excitatory claustro-cortical projections preferentially synapse onto cortical inhibitory interneurons (Jackson, 2018; Smith et al., 2019; Narikiyo et al., 2020). This extensive connectivity positions the CLA as a central network hub that coordinates information exchange across the cerebral cortex and contributes to multimodal sensory integration (Pearson et al., 1982; Smith et al., 2019).

Anatomical and connectivity studies have shown that the CLA receives strong inputs from serotonergic (5-HT) neurons of dorsal raphe nucleus (DRN) (Zingg et al., 2018; Vertes, 1991; Peyron et al., 1998), and expresses high levels of 5-HT receptors, including 5-HT1A, 5-HT1B, 5-HT1F, 5-HT2A and 5-HT2C receptors (Bruinvels et al., 1994; Pompeiano et al., 1994; Wright et al., 1995; Kinsey et al., 2001). Of particular interest is the high expression of 5-HT2ARs in the CLA, which are a target of serotonergic psychedelics, such as lysergic acid diethylamide (LSD) and psilocybin. Canonically, these compounds act as full or partial agonists at 5-HT2ARs and induce an atypical state of consciousness characterized by altered sensory perception and executive functions (Carhart-Harris et al., 2016; Preller et al., 2018; Vollenweider and Preller, 2020). Importantly, the hallucinogenic effects of serotonergic psychedelics are mediated, at least in part, through alterations in CLA function (Barrett et al., 2020; Doss et al., 2022; Reiff et al., 2020). Despite the established role of CLA in mediating the behavioral effects of serotonergic psychedelics, the mechanisms by which 5-HT and its receptors, including 5-HT2Ars, modulate the function of CLA neurons remain ill-defined. Using *ex vivo* whole-cell recordings from adult male rats, we identified four types of CLA neurons that exhibit distinct firing patterns and differential responses to 5-HT application. Specifically, 5-HT induces either membrane hyperpolarization or depolarization mediated by activation of 5-HT1ARs and 5-HT2ARs, respectively. In addition, we found that activation of 5-HT2ARs reduces the strength of glutamate synapses in the CLA by inhibiting glutamate release. As such, the present study unravels multifaceted mechanisms by which 5-HT signaling regulates the neuronal function and synaptic transmission in CLA neurons.

## Materials and methods

### Animals

All experimental procedures used in this study were approved by the University at Buffalo Institutional Animal Care and Use Committee (IACUC) and were conducted in accordance with the National Institutes of Health Guide for the Care and Use of Laboratory Animals. The experiments are reported in compliance with the ARRIVE guidelines. Male Sprague-Dawley rats were purchased from Envigo (Envigo, Indianapolis, IN, USA). Animals were housed in pairs, with a 12/12 light/dark cycle and ad libitum food and water. Rats were allowed to acclimate to the animal facility for a week before experiments began.

### Brain slice preparation

Brain slices containing the CLA were prepared from male Sprague-Dawley rats (4–8 weeks old) using a standard procedure. Briefly, rats were deeply anesthetized with isoflurane and decapitated using guillotine, the forebrain region containing the CLA was isolated and sliced (350 μm) using a vibratome (Leica VT1200S; Leica Biosystem, St Louis, MO, USA) in ice-cold cutting solution of the following composition (in mM): 110 Choline-Cl; 2.5 KCl; 0.5 CaCl_2_; 7 MgSO_4_; 1.25 NaH_2_PO_4_; 26.2 NaHCO_3_; 11.6 sodium L-ascorbate; 3.1 sodium pyruvate, 25 glucose and saturated with 95% O_2_/5% CO_2_. Slices were first incubated at 35 °C in the cutting solution for 15 min and then 30 min in regular artificial cerebrospinal fluid (ACSF) containing (in mM): 119 NaCl; 2.5 CaCl_2_; 1.3 MgSO_4_; 1 NaH_2_PO_4_; 26.2 NaHCO_3_; 11 glucose and continuously bubbled with a mixture of 95% O_2_/5% CO_2_. After incubation, slices were maintained at room temperature for full recovery at least one hour before electrophysiological recordings.

### Whole-cell recordings

Following recovery, slices were transferred to a recording chamber (Warner Instruments, Hamden, CT, USA) mounted on a fixed upright microscope and continuously perfused (2–3 ml/min) with regular ACSF solution saturated with 95% O_2_/5% CO_2_ and heated to 30 °C ± 1 °C using a solution heater (Warner Instruments, Hamden, CT, USA). CLA neurons were visualized using Olympus BX51 microscope equipped with a 40× water-immersion objective, differential interference contrast (DIC) and epifluorescence imaging systems. Somatic whole-cell recordings from CLA neurons were performed using patch electrodes with tip resistance of 3–5 MΩ, filled with an internal solution containing (in mM): 120 potassium gluconate; 10 KCl; 10 Na_2_-phosphocreatine; 10 HEPES; 1 MgCl_2_; 1 EGTA; 2Na_2_-ATP; 0.25 Na-GTP; pH = 7.3 adjusted with KOH and, osmolality of 280–290 mOsm/L.

Evoked excitatory postsynaptic currents (eEPSCs) were induced using patch pipettes (2–4 MΩ) filed with ACSF and placed 100–200 μm dorsal to the recorded neurons. Excitatory post synaptic currents were evoked by extracellular electrical stimulation (i.e., single square-pulses, duration of 100–200 μs) delivered at 0.1 Hz and recorded from claustrum neurons voltage-clamped at −70 mV in the presence of picrotoxin (PTX) 100 μM to block GABA_*A*_ receptors. The intensity of the electrical stimulation was adjusted to evoke stable baseline amplitude around 75% of the maximal EPSCs. To determine the paired-pulse ratio (PPR), a pair of EPSCs were evoked with an inter-stimulus interval of 50 ms. Spontaneous excitatory postsynaptic currents (sEPSCs) were recorded in the presence of PTX, whereas miniature EPSCs (mEPSCs) were recorded in the presence of PTX and tetrodotoxin (TTX, 1 μM) to block GABAA receptors and voltage-gated sodium channels, respectively. The neuron’s input and access resistance (10–20 mΩ) were continuously monitored throughout the experiment by 5 mV hyperpolarizing voltage steps (500 ms duration). Recordings were discarded when the input and series resistance changed by more than 20%. Membrane voltage and current were amplified using Axoclamp 2B, digitized with Digidata 1550B and acquired using the pClamp 10.2 software (Molecular Devices, Union City, CA, USA).

### Immunohistochemistry

For biocytin labeling, whole-cell recordings of CLA neurons were performed using K-Gluconate based internal solution containing 0.1% biocytin. After 45–60 min of intracellular diffusion of biocytin, slices were stored in 4% PFA at 4 °C for 24 h, then transferred to PBS and washed 3 times for 5 min. After washing, slices were permeabilized with 1% Triton X100/PBS at 4 °C for 48 h and then incubated in Streptavidin Alexa 488 (ThermoFisher, Waltham, MA, USA) diluted to 1:1000 in 0.3% Triton X100/PBS at 4 °C for 24 h. After a final series of three washes with PBS, slices were mounted onto glass microscope slides for imaging. For 5-HT2AR immunostaining, forebrain coronal sections (30 μm) containing the CLA were incubated with rabbit anti 5-HT2AR (1:400, Immunostar, Hudson, WI, USA) and secondary antibody Alexa Fluor 594 donkey anti-rabbit (1:1000, Jackson ImmunoResearch Labs, West Grove, PA, USA).

### Data analysis

Evoked excitatory postsynaptic currents (eEPSCs) were analyzed using Clampfit software (Molecular Device, San Jose, CA, USA). The amplitude of eEPSCs was determined by measuring the average current over a 2 ms window centered on the peak of eEPSC, subtracted from the baseline current. To assess the effects of 5-HT and 5-HT2AR on the amplitude of eEPSCs, the amplitude of EPSC was normalized to the mean baseline amplitude recorded for at least 5 min before drug application. For paired-pulse experiments the paired-pulse ratios (PPR = eEPSC_2_/eEPSC_1_) were averaged for at least 30 consecutive trials before and during drug application. Statistical analysis was performed using Origin 2019b software (Origin Lab Co., Northampton, MA, USA). Miniature excitatory postsynaptic currents (mEPSCs) were analyzed with Mini Analysis Software (Synaptosoft, Decatur, GA, USA). The synaptic events were selected using amplitude threshold of 5 pA, rise time (1 ms), and area threshold (30 fC). All selected events were further visually inspected to prevent noise from compromising the analysis. The data distribution was tested for normality using Shapiro-Wilk test. A parametric paired *t*-test was used for normally distributed data and within-group. For comparison between groups, independent *t*-test and analysis of variance (ANOVA) using *post hoc* Tukey tests were used. Statistical analysis of sEPSCs and mEPSCs was conducted using Kolmogorov–Smirnov tests (K–S test). The results in the text and figures are expressed as mean ± SEM and significance was set at α = 0.05.

### Drugs and chemicals

Chemicals, salts, and fluoroacetate were obtained from Fisher Scientific (Pittsburgh, PA, USA). N-(Piperidin-1-yl)-5-(4-iodophenyl)-1-(2,4-dichlorophenyl)-4-methyl-1H-pyrazole-3-carboxamide (AM251), Picrotoxin, TCB-2, U73122, 5-HT, mesulergine, MDL100,907 were purchased from Tocris Biosciences (Minneapolis, MN, USA) and guanosine 5′-[β-thio] diphosphate (GDPβ-S) from Sigma-Aldrich (Saint Louis, MO, USA). Tetrodotoxin (TTX) citrate was purchased from HelloBio (Princeton, NJ, USA) and RuBi5-HT from Abcam (Waltham, MA, USA). All antibodies were purchased from ThermoFisher Invitrogen (Waltham, MA, USA), except 5-HT2AR antibody, which was obtained from Immunostar (Hudson, WI, USA).

## Results

### Electrophysiological characterization of CLA neurons

*Ex vivo* whole-cell recordings from CLA neurons revealed four electrophysiologicaly distinct groups of neurons based on their voltage responses to constant depolarizing and hyperpolarizing current injections. Regular spiking type I neurons (RSI) exhibit a noticeable voltage sag induced by strong membrane hyperpolarization and regular firing activity with very weak spike accommodation in response to long (600 ms) suprathreshold depolarizing current pulses (Figure 1A1). These neurons have a mean resting membrane potential (RMP) of −71.59 ± 0.65 mV (Figure 1B1, *n* = 28, 46% of sampled neurons), an input resistance of 149.04 ± 10.27 MΩ (Figure 1B2, *n* = 28 neurons) and a rheobase current of 136.60 ± 12.76 pA (Figure 1B3, *n* = 28 neurons). The maximum firing frequency induced by a constant suprathreshold depolarizing current (600 ms) pulses is 13.52 ± 2.6 Hz (*n* = 23 neurons). Regular spiking type II (RSII) neurons exhibit a fast afterdepolarization potential (fADP), which triggered a doublet of action potentials (Figure 1A2). These neurons show similar firing frequency (13.33 ± 1.8Hz, *n* = 13, 21% of sampled neurons) and mean RMP (−67.61 ± 1.81 mV, *n* = 13 neurons, *p* > 0.05 vs. RSI, Figure 1B1), but significantly higher rheobase current (204.16 ± 22.17 pA, *n* = 12 neurons, *p* < 0.05 RSII vs. RSI, Figure 1B3) and lower input resistance (102.55 ± 8.44 MΩ, *n* = 12 neurons, *p* < 0.01, RSII vs. RSI, Figure 1B2) than RSI neurons. Strong adapting (SA) neurons respond to suprathreshold depolarizing pulses with strong spike adaptation and very low firing activity (Figure 1A3). These neurons have a mean RMP of −72.18 ± 1.09 mV (Figure 1B1, *n* = 12 neurons), a significantly higher rheobase current of (193.84 ± 26.22 pA, *p* < 0.05 SA vs. RSI, *n* = 13 neurons Figure 1B3), and lower mean input resistance (118.56 ± 9.93 MΩ, *p* < 0.05, SA vs. RSI, *n* = 11 neurons, Figure 1B2) than RSI neurons. The fourth group which represent a very small fraction of sampled neurons, exhibit a fast-spiking pattern (Figure 1A4), with a mean max firing frequency of 60 ± 6.5 Hz. The neurons exhibit a comparable average RMP (−71.03 ± 2.80 mV, Figure 1B1, *n* = 7 neurons) to other neurons, but significantly lower input resistance than RSI and SA neurons (104.85 ± 15.15 MΩ, *p* < 0.05, *n* = 7 neurons, Figure 1B2) and significantly lower rheobase current (137.14 ± 26.06 pA, *p* < 0.05, *n* = 7 neurons, Figure 1B3) that RSII and SA neurons.

**FIGURE 1:**
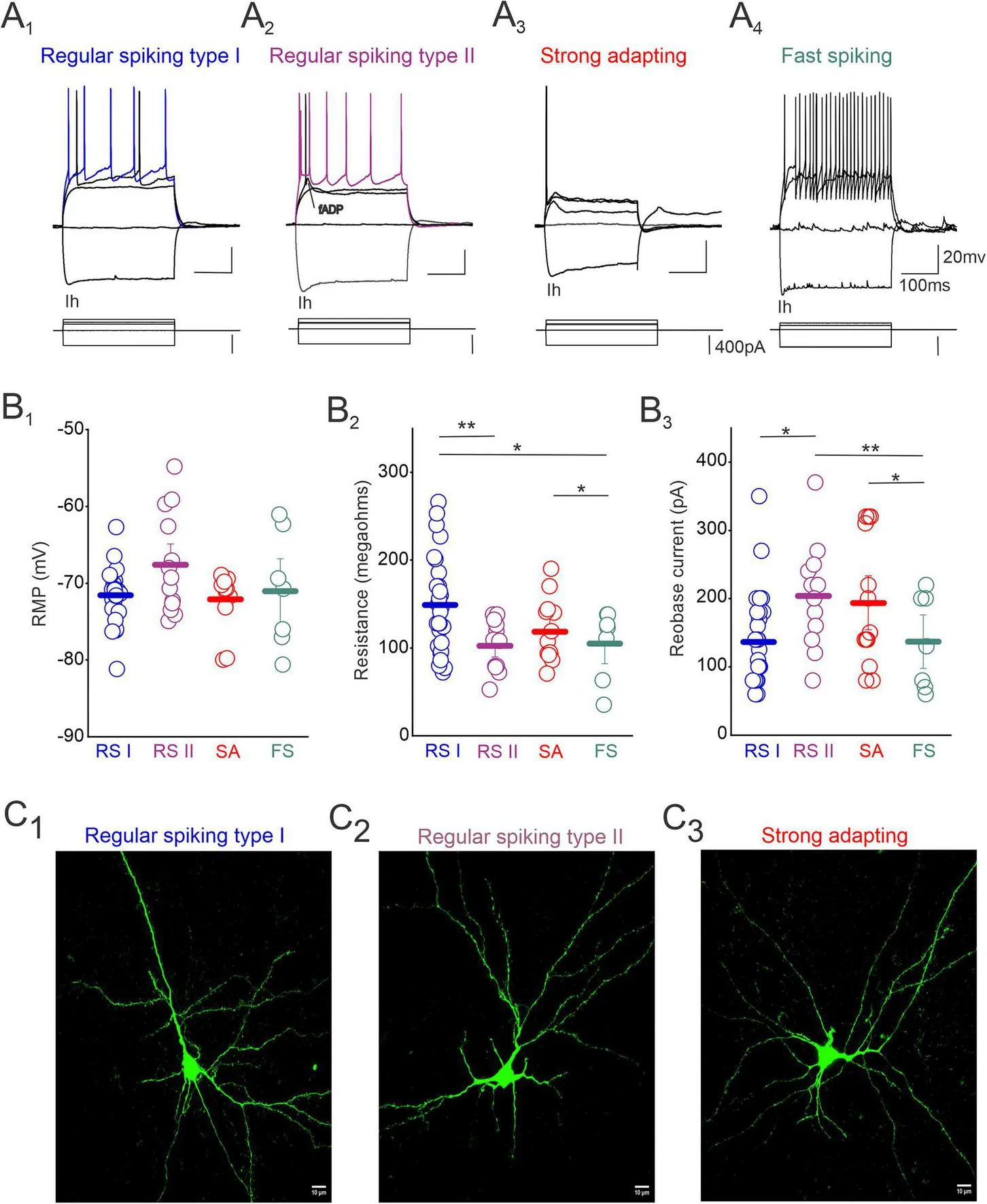
Intrinsic electrophysiological and morphological properties of the main CLA neurons. **(A_1_–A_3_)** Representative membrane potential responses to 600 ms constant depolarizing and hyperpolarizing current pulses depicting, regular spiking type I [RSI, **(A_1_)**], type II [RSII, **(A_2_)**], strong adapting [SA, **(A_3_**), and fast spiking [FS, **(A_4_)**] neurons. **(B_1_–B_3_)** Average resting membrane potential **(B_1_)**, membrane resistance **(B_2_)**, and rheobase current **(B_3_)** of CLA neurons. **(C_1_–C_3_)** Representative images of biocytin labeled RSI **(C_1_)**, RSII **(C_2_)**, and SA **(C_3_)** CLA neurons. Scale bars: 10 μm. *p < 0.5; **p < 0.01.

Previous morphological studies have reported that CLA neurons are morphologically heterogeneous with polygonal, triangular, round or fusiform cell bodies (Shibuya and Yamamoto, 1998; Rahman and Baizer, 2007; White and Mathur, 2018) and exhibit either spiny or aspiny dendrites (White and Mathur, 2018). Therefore, we next examined whether RSI, RSII, and SA CLA neurons correspond to distinct morphological cell types. Biocytin labeling and *post hoc* immunostaining of 12 recorded neurons showed that RSI, RSII, and SA neurons were spiny neurons (Figures 1C1–C3). The soma of RSI neurons exhibited a pyramidal-like shape with an apical dendrite (Figure 1C1, *n* = 4 neurons), whereas the cell bodies of RSII (Figure 1C2, *n* = 4 neurons) and SA neurons (Figure 1C3, *n* = 4 neurons) were triangular with multiple dendrites arising from the soma.

### Post-synaptic effects of 5-HT on CLA neurons

Examination of the effects of 5-HT on the excitability of CLA neurons revealed that bath application of 5-HT (30 μM) induced membrane depolarization in most recorded RSI and RSII neurons (Figure 2A, *N* = 12/23). In a few RSI and RSII neurons, 5-HT also induced membrane hyperpolarization (Figure 2B, *N* = 4/23), whereas no effect was observed in the remaining neurons (*n* = 7/23). In contrast, all SA neurons tested were hyperpolarized by 5-HT (Figure 2C, *N* = 5 neurons). Using caged-serotonin (RuBi-5-HT) we examined the effects of a transient increase in extracellular 5-HT on the RMP of CLA neurons. We found that brief uncaging of 5-HT (λ = 450 nm, 5 s) induced either membrane depolarization (Figures 2C–E, ΔVm = 4.08 ± 0.57 mV, *n* = 8/11 neurons), or membrane hyperpolarization (Figures 2D, E, ΔVm = 3.90 ± 0.24 mV, *n* = 8/12 neurons), in RSI and RSII neurons. The depolarization response was blocked by the 5-HT2AR antagonist MDL 100907 (3 μM) (Sorensen et al., 1993) (Figure 2C, *N* = 6 neurons), whereas the hyperpolarization was abolished by the 5-HT1AR antagonist WAY100635 (3 μM) (Fletcher et al., 1996; Figure 2D, *N* = 4 neurons). Taken together, these results indicate that 5-HT exerts opposing effects on the RMP of RSI and RSII neurons through activation of postsynaptic 5-HT2A and 5-HT1A receptors.

**FIGURE 2:**
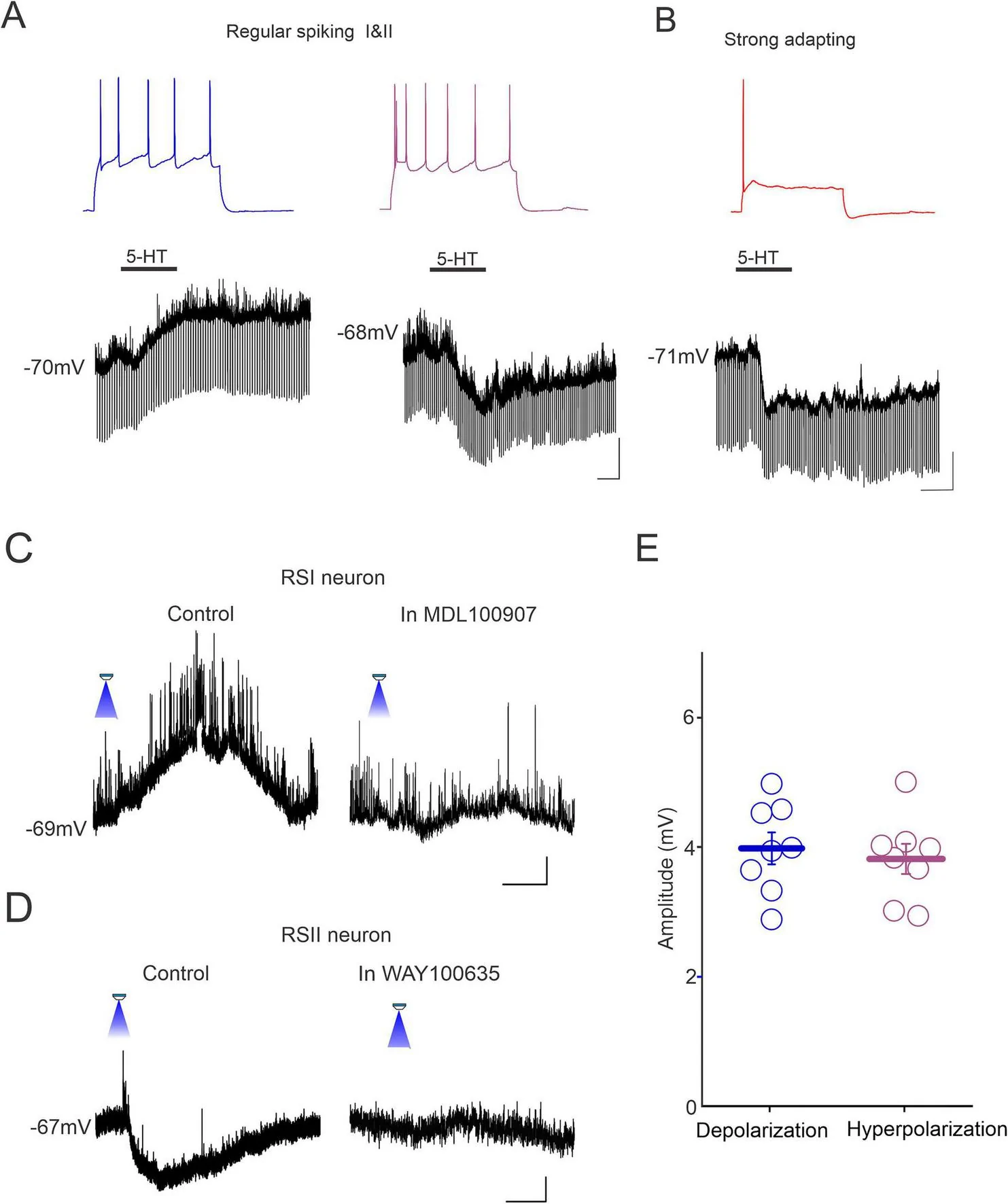
5-HT depolarizes and hyperpolarizes CLA neurons by activation of 5-HT2AR and 5-HT1AR, respectively. **(A)** Voltage traces representing the 5-HT induced membrane depolarization (left lower panel) and hyperpolarization (right lower panel) of RSI and RSII CLA neurons. Upper panel illustrates the firing pattern of RSI and RSII neurons. **(B)** Voltage traces illustrating the 5-HT induced membrane hyperpolarization of a SA neuron (lower panel). Upper panel firing pattern of the corresponding SA neuron. **(C)** Representative membrane depolarization induced by 5-HT uncaging in control (left trace) and in the presence of MDL100907 (right trace). Scale bars: 5 mV, 2 min. **(D)** Membrane hyperpolarization of CLA neurons induced by 5-HT uncaging in control (left trace) and in the presence of WAY100635 (right trace). Scale bars: 2.5 mV, 5 s. **(E)** Averaged membrane depolarization and hyperpolarization of CLA neurons induced by 5-HT uncaging.

### Serotonin inhibits CLA glutamate synapses through 5-HT2ARs

In addition to the postsynaptic effects, we also assessed the impact of 5-HT on glutamatergic synaptic transmission onto CLA neurons. We found that 5-HT (30 μM) significantly decreased the amplitude of evoked excitatory postsynaptic currents (eEPSCs) (49.4% ± 6.4% of baseline, *n* = 8, *p* = 0.0151, 5-HT vs. baseline, Figure 3A1). This effect was associated with an increased paired-pulse ratio (PPR baseline = 0.95 ± 0.08, PPR 5-HT = 1.25 ± 0.08, *n* = 8, *p* = 0.0075, Figure 3A2) and coefficient of variation (CV baseline = 0.14 ± 0.02, CV 5-HT = 0.25 ± 0.05, *n* = 8, *p* = 0.034, Figure 3A3), thereby indicating that 5-HT reduces the strength of glutamate synapses by reducing glutamate release. To further support this notion, we first tested whether the reduction of glutamatergic transmission is caused by inhibition of local network activity. To that end, we assessed the effect of 5-HT on spontaneous excitatory postsynaptic currents (sEPSCs). We found that 5-HT significantly reduced the frequency of sEPSCs (control = 7.97 ± 0.68 Hz, 5-HT = 3.96 ± 0.48 Hz, *n* = 9, *p* = 0.0085, Figure 3B3), leading to significant rightward shift of the cumulative distribution of the inter-events intervals (Figure 3B2, K-S test, *p* < 0.05, *n* = 9 neurons). This effect was not associated with a significant alteration in the amplitude of sEPSCs (Control = 14.10 ± 1.11 pA, 5-HT = 14.08 ± 1.11 pA, *n* = 9 neurons, *p* = 0.124, Figure 3B4). We next examined the impact of 5-HT on the frequency and amplitude of mEPSCs, a direct measure of action potential independent quantal glutamate release and found that 5-HT significantly reduced the frequency (Control = 3.85 ± 0.58 Hz; 5-HT = 1.75 ± 0.38 Hz, *n* = 10 neurons, *p* = 0.025, Figures 3C1–C3) of mEPSC. Collectively, these results demonstrate that 5-HT inhibits excitatory synaptic drive to CLA neurons by reducing the probability (Pr) of glutamate release.

**FIGURE 3:**
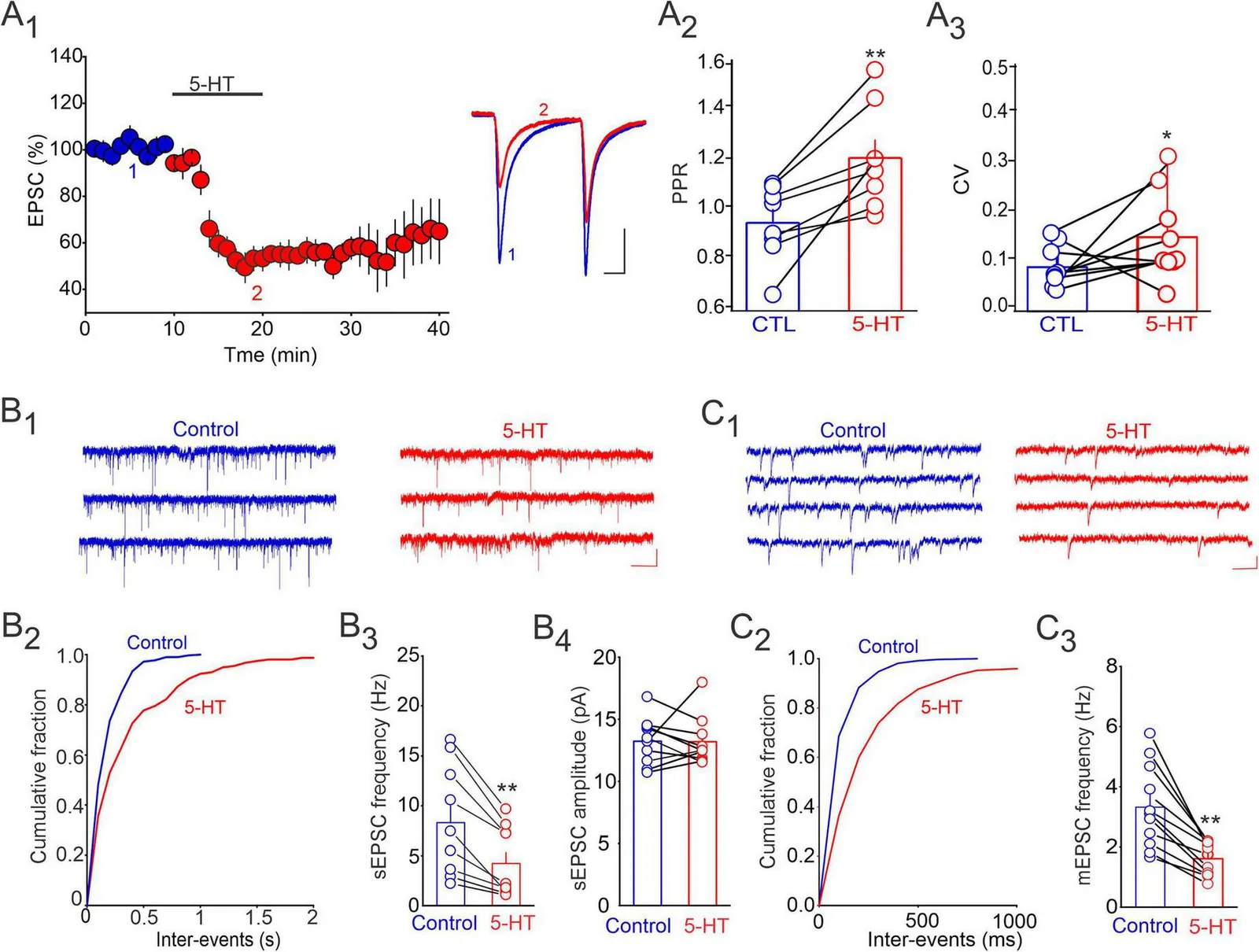
5-HT inhibits CLA glutamate synapses by reducing glutamate release. **(A_1_)** Time course and magnitude of 5-HT-induced inhibition of eEPSC amplitude (left panel). Right panel illustrates averaged eEPSC traces taking in the absence and presence of 5-HT. Scale bars: 50 pA, 10 ms. **(A_2_)** Averaged PPR of eEPSCs obtained in the absence and presence of 5-HT. **(A_3_)** Averaged CV of eEPSCs determined in the absence and presence of 5-HT. **(B_1_)** Sample current recordings of sEPSCs in control (left traces) and in the presence of 5-HT (right traces). Scale bars: 5 pA, 1 s. **(B_2_)** Cumulative distribution of sEPSC frequency obtained in control and in the presence of 5-HT. **(B_3_)** Averaged sEPSC frequency recorded in control and in the presence of 5-HT. **(B_4_)** Averaged sEPSC amplitude obtained in control and in the presence of 5-HT. **(C_1_)** Sample current traces of mEPSCs collected in control and in the presence of 5-HT. Scale bars: 5 pA, 500 ms. **(C_2_)** Cumulative distribution of mEPSC frequency obtained in control and in the presence of 5-HT. **(C_3_)** Averaged mEPSC frequency recorded in control and in the presence of 5-HT. *p < 0.5.

Results from previous studies showed that among various 5-HT receptors, the CLA expresses high levels of 5-HT2Rs, including 5-HT2ARs and 5-HT2CRs (Pompeiano et al., 1994; Wright et al., 1995; Erwin et al., 2021). Therefore, we tested whether these receptors mediate the inhibition of CLA glutamate synapses. We found that in the presence of mesulergine (3 μM), a non-selective 5-HT2R antagonist (Hoyer et al., 1994), bath application of 5-HT (30 μM) did not induce inhibition but rather produced a significant potentiation of the amplitude of eEPSC (In control: 45.65% ± 4.6% of baseline, In mesulergine: 145.7% ± 25.5% of baseline, *n* = 7, *p* = 0.015, 5-HT vs. baseline, Figure 4A). The observed 5-HT-induced potentiation in the presence of mesulergine suggest that blocking 5-HT2Rs unmask a serotonin response that may be mediated by other non-5-HT2Rs expressed in the CLA. Treatment of brain slices with the selective 5-HT2AR antagonist MDL100907 (3 μM) blunted the inhibition of eEPSC amplitude induced by 5-HT (30 μM) (In control: 51.89% ± 4.56% of baseline, In MDL100907: 105.89% ± 6.5% of baseline, *n* = 10, *p* > 0.05, Figure 4B). In addition, compared to control condition (Figure 2C3), treatment with MDL 100907 prevented the 5-HT-induced reduction in mEPSC frequency (MDL100907: 3.35 ± 0.65 Hz; MDL100907+5-HT: 3.79 ± 0.75 Hz, *n* = 11 neurons, *p* > 0.05, Figures 4C1, C2), suggesting that 5-HT2ARs mediate the 5-HT-induced inhibition of CLA glutamate synapses. To further confirm this conclusion, we examined the effects of TCB2, a high-affinity and potent 5-HT2AR agonist (McLean et al., 2006). As illustrated in Figure 4D1, administration of TCB2 (3 μM) reduced the amplitude of eEPSCs (In control: 57.85% ± 4.65% of baseline, *n* = 9 neurons, *P* = 0.0089, TCB2 vs. baseline), an effect that was readily blocked by the 5-HT2AR antagonist MDL100909 (3 μM) (In MDL100907: 96.89% ± 8.69% of baseline, *n* = 9 neurons, *p* = 0.0789, baseline vs. TCB2, Figure 4D1). The TCB2-induced inhibition of eEPSC amplitude was associated with increased PPR (Control: 0.94 ± 0.09, TCB2: 1.36 ± 0.12, *n* = 5 neurons, *p* = 0.026, Figure 4D2). Activation of 5-HT2ARs with TCB2 (3 μM) also reduced the frequency (Control: 2.98 ± 0.59 Hz, TCB2: 1.36 ± 0.26 Hz, *p* = 0.034, *n* = 11 neurons, Figures 4E1, E2), but not the amplitude of mEPSCs (Control: 10.61 ± 0.8 pA, TCB2: 10.82 ± 0.99 pA, *p* = 0.642, *n* = 11 neurons, Figures 4E4, E5). Taken together, these results indicate that 5-HT2ARs reduce the strength of CLA glutamate synapses by inhibiting glutamate release.

**FIGURE 4:**
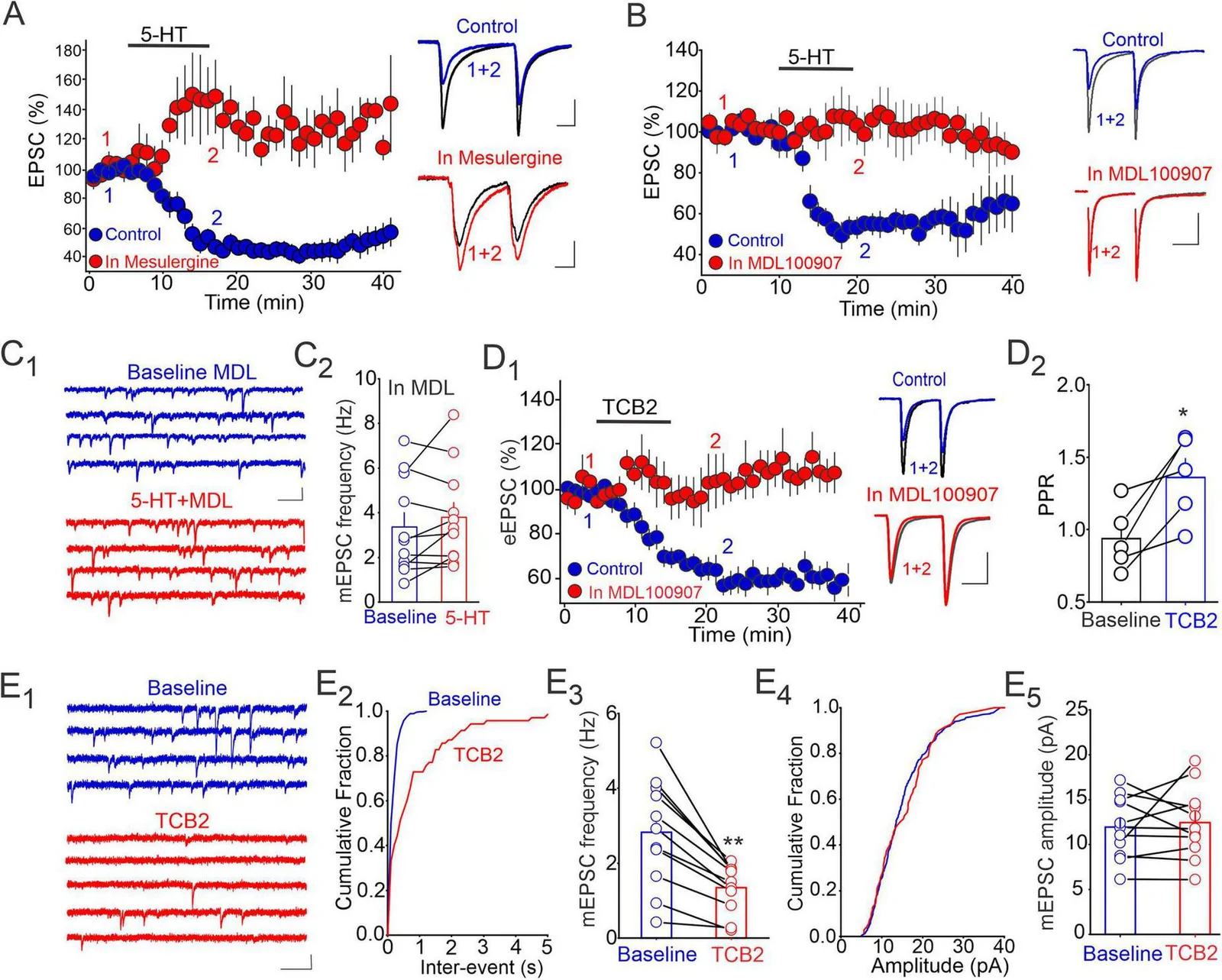
5-HT reduces the strength of CLA glutamate synapses via activation of 5-HT2ARs. **(A)** Time course and magnitude of the effects of 5-HT (30 μM) on eEPSCs amplitude in control and in the presence of 5-HT2R antagonist, mesulrgine (left panel). Right panel illustrates average eEPSC traces sampled at time point indicated by number in left panel. Scale bars: 50 pA, 20 ms. **(B)** Blockade of 5-HT2ARs abolishes 5-HT-induced inhibition of eEPSC amplitude. Left panel illustrates time course and magnitude of 5-HT-induced inhibition of eEPSC amplitude in control and in the presence of 5-HT2AR antagonist, MDL100907. Right panel depicts averaged and superimposed eEPSC traces collected at time point indicated by numbers in left panel. Scale bars: 50 pA, 20 ms. **(C_1_)** Current recordings depicting the effects of 5-HT on mEPSCs in the presence of MDL 100907. Scale bars: 5 pA, 500 ms. **(C_2_)** Average mEPSC frequency recorded in MDL 100907 in the absence and presence of 5-HT. **(D_1_)** Time course and magnitude of the inhibition of eEPSC amplitude induced by TCB2 (3 μM) in control and in the presence of MDL100907 (1 μM) (left panel). Right panel illustrates averaged superimposed eEPSC traces sampled at time points indicated by numbers in the left graph. Scale bars: 50 pA, 20 ms. **(D_2_)** Averaged PPR of eEPSCs obtained in control and in the presence of TCB2. **(E_1_)** Sample recordings of mEPSCs in control and in the presence of TCB2. Scale bars: 5 pA, 500 ms. **(E_2_)** Cumulative distribution of mEPSC frequency in control and in the presence of TCB2. **(E_3_)** Averaged mEPSC frequency obtained in control and in TCB2. **(E_4_)** Cumulative distribution of mEPSC amplitude recorded in control and in the presence of TCB2. **(E_5_)** Averaged mEPSC amplitude in control and in the presence of TCB2. *p < 0.5; **p < 0.01.

### The inhibition of glutamate synapses is not mediated by postsynaptic 5-HT2ARs and PLC signaling cascade

In the brain, 5-HT2ARs are mainly located on the cell body, dendrites and neuronal axons (Cornea-Hébert et al., 1999; Jakab and Goldman-Rakic, 1998; Willins et al., 1997). To assess the distribution of 5-HT2ARs in the CLA, we performed immunohistochemical labeling of these receptors. Consistent with earlier receptor binding (Rioux et al., 1999), immunohistochemical (Pompeiano et al., 1994), and transcriptomic studies (Erwin et al., 2021), our results showed a dense immunolabelling of 5-HT2ARs in the deep layers of the cerebral cortex and CLA (Figure 5A1). In the CLA, the intense 5-HT2AR staining was observed around the cell bodies (Figure 5A2) of neurons and along their neuronal processes (Figure 5A2). To examine whether postsynaptic 5-HT2ARs mediate the inhibition of glutamatergic synaptic transmission, we tested the effect of blocking postsynaptic G-protein signaling on TCB2-induced reduction of eEPSC amplitude. We found that postsynaptic intracellular application of GDP_β_S, which blocked TCB2-induced inward shift of the holding current (GTP ΔIm = −105.2 ± 6.8 pA; GDPβS = −10.5 ± 4.6 pA, *n* = 8 neurons, *p* < 0.05), a response signaled by postsynaptic 5-HT2ARs, did not alter the magnitude of TCB2-induced inhibition of eEPSC amplitude (GTP: 60.35% ± 3.65% of baseline, GDPβS: 58.75% ± 5.45% of baseline, *n* = 8 neurons, *p* > 0.05 unpaired *t*-test, Figure 5B. In addition, blocking postsynaptic G-protein signaling did not prevent the increase in PPR induced by TCB2 (Baseline control: 0.95 ± 0.06, TCB2 control: 1.18 ± 0.09, *n* = 7 neurons, *p* < 0.05, Baseline control vs. TCB2 control, Figure 5C, Baseline GDP_β_S: 0.82 ± 0.046, TCB2 GDP_β_S: 0.925 ± 0.038, *n* = 7 neurons, *p* < 0.05, Baseline GDP_β_S vs. TCB2 GDP_β_S, Figure 5D). However, the magnitude of TCB2-induced increase in PPR obtained with GDPβS was less profound than with GTP. These results exclude the involvement of postsynaptic 5-HT2ARs in mediating TCB2-induced of CLA glutamate synapses.

**FIGURE 5:**
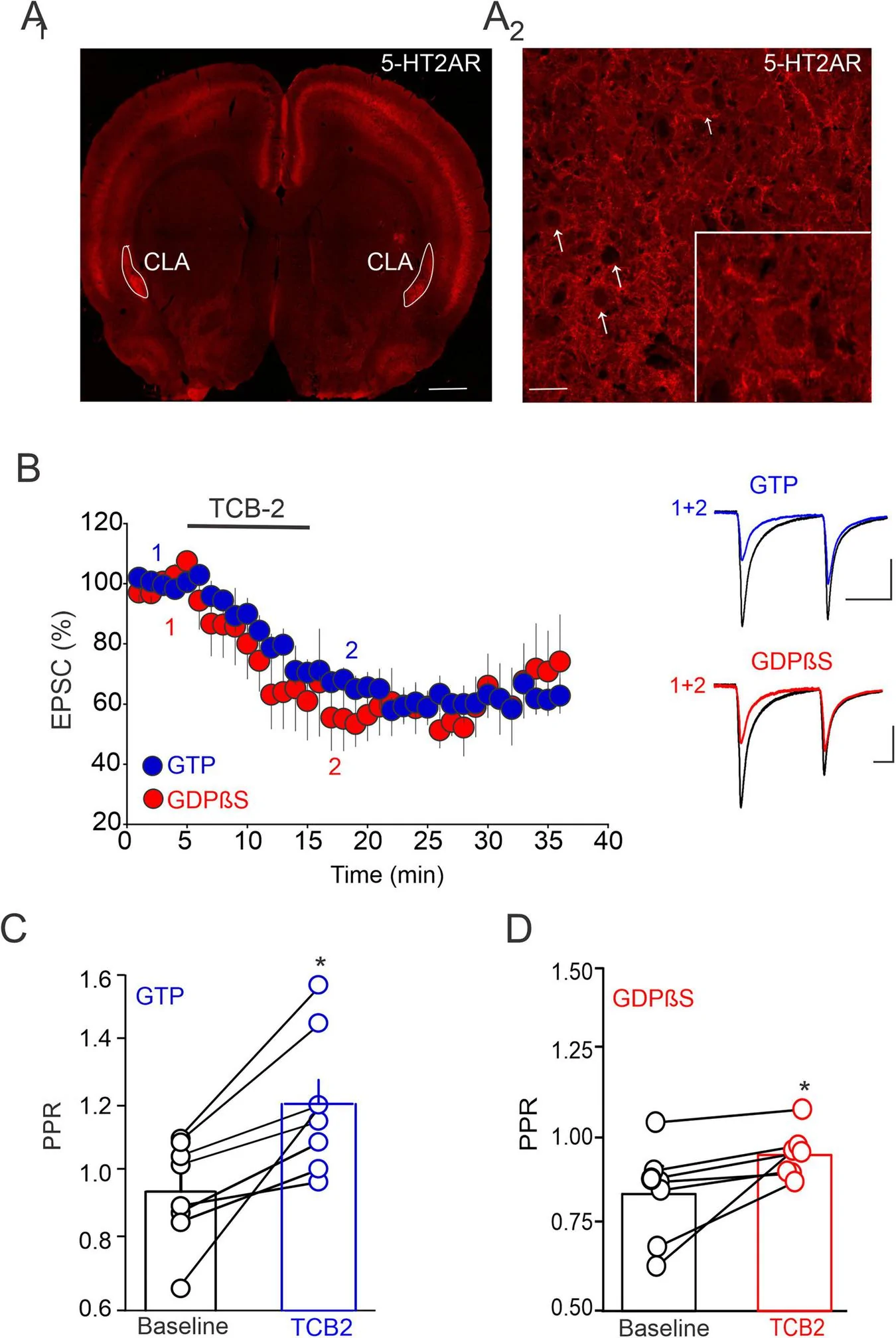
The 5-HT-induced inhibition of glutamate synapses is not mediated by postsynaptic 5-HT2ARs. **(A_1_)** Low magnification image of 5-HT2AR immunostaining of rat cortical and subcortical areas, including CLA. **(A_2_)** Higher magnification of 5-HT2ARs fluorescence in the CLA. Arrows indicate somatic and membrane labeling of 5-HT2ARs. **(B)** Left panel illustrates a summary of the time course and magnitude of TCB2-induced reduction of eEPSC amplitude recorded with control and GDPβS containing internal solution Right panel depicts averaged and superimposed eEPSC traces collected at time points indicated by numbers in the left graph. **(C)** Averaged PPR of eEPSCs recorded with control internal solution at baseline and in presence of TCB2. **(D)** Averaged PPR of eEPSCs recorded with internal solution containing GDPβS at baseline and in presence of TCB2. Scale bars: 50 pA, 20 ms. *p < 0.05.

Serotonin 5-HT2ARs are canonically coupled to G_*q/11*_ type G-proteins and activate phospholipase C (PLC) downstream signaling cascade (Roth et al., 1998; Marin et al., 2012). Therefore, we tested the role of PLC in mediating the effects of TCB2 on eEPSCs. We found that the magnitude of TCB2-induced inhibition of eEPSC amplitude obtained in slices treated with the PLC inhibitor U73122 (10 μM) was comparable to that obtained in slices treated with the inactive analog U73343 (U73343: 62.3% ± 2.5% of baseline; U73122: 52.56% ± 8.9% of baseline, *n* = 8 neurons, *p* = 0.456, U73343 vs. U73122, Figure 6A). Such a result suggests that PLC signaling cascade may not be involved in mediating the 5-HT2AR-induced inhibition of CLA glutamate synapses. Activation of 5-HT2ARs can also increase the synthesis and release of endocannabinoids (eCBs) (Best and Regehr, 2008; Parrish and Nichols, 2006), which engages presynaptic cannabinoid type 1 receptors (CB1Rs) and inhibit synaptic transmission (Haj-Dahmane and Shen, 2005; Oubraim et al., 2023). Therefore, we tested this possibility by assessing the impact of blocking CB1Rs on the magnitude of TCB2-induced reduction of eEPSC amplitude. The results revealed that treatment of CLA slices with AM251 (3 μM), a CB1R antagonist/reverse agonist, did not block TCB2-induced inhibition of eEPSC amplitude (Control: 52.5% ± 5.3% of baseline, AM 251: 45.68% ± 4.9% of baseline, *n* = 9 neurons; *p* = 0.556 control vs. AM251, Figure 6B). Taken together, these results suggest that activation of presynaptic 5-HT2ARs reduces glutamatergic synaptic transmission in the CLA through PLC and eCB independent mechanisms.

**FIGURE 6:**
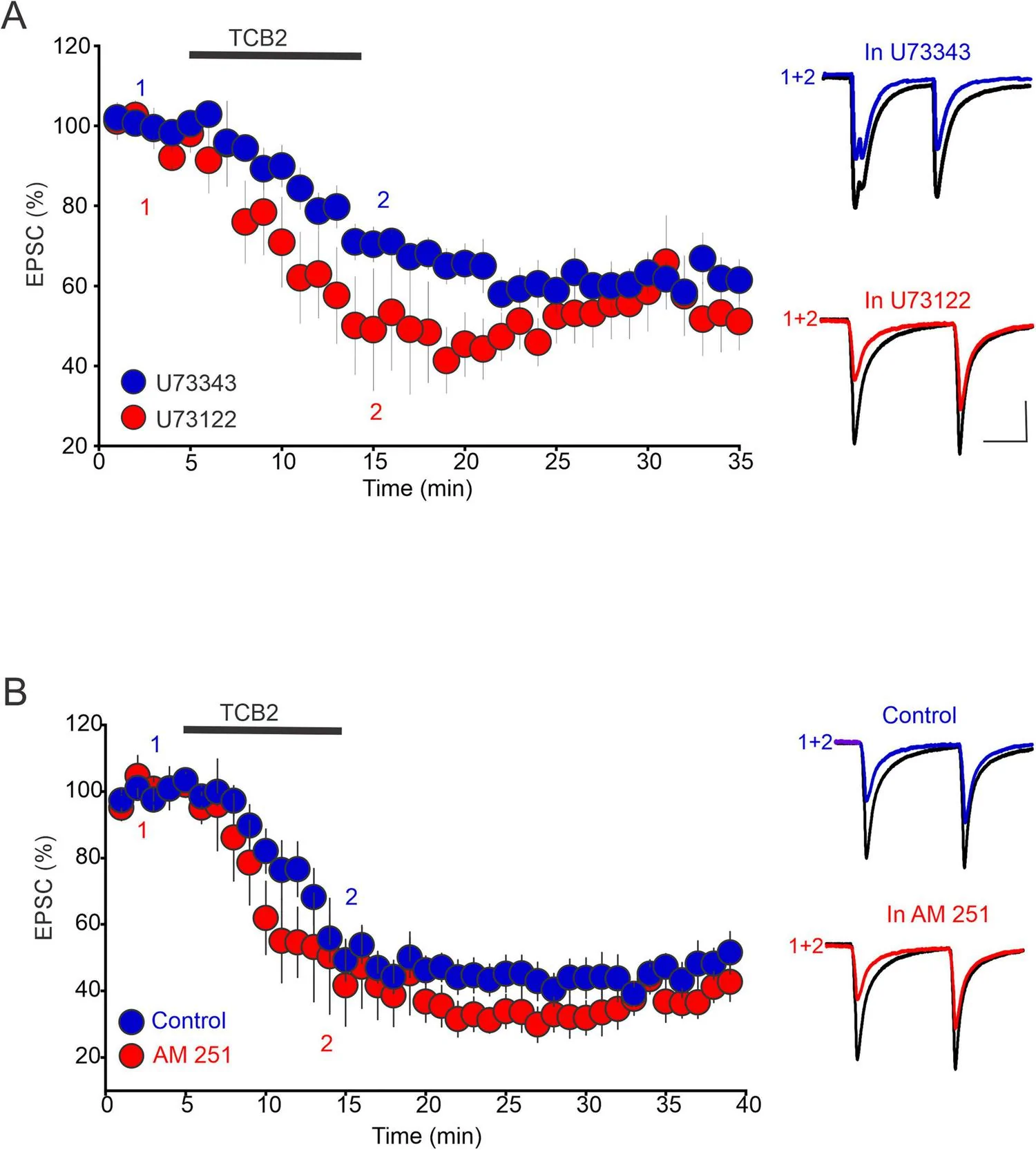
The 5-HTAR-induced inhibition of glutamate synapses is not mediated by PLC and eCB signaling. **(A)** Left graph illustrate the course and magnitude of the inhibition of eEPSC amplitude induced by TCB2 (3 μM) in slices treated with the PLC inhibitors U73122 (10 μM) and its inactive analog U73343 (10 μM). Right panel illustrates averaged and superimposed eEPSC traces taken by numbers indicated in the left graph. Scale bars: 50 pA, 20 ms. **(B)** Time course and magnitude of the inhibition of eEPSC amplitude induced by TCB2 in control slices and slices treated with the CB1R antagonist AM251 (10 μM). Right panel illustrates averaged and superimposed eEPSC traces taken by numbers indicated in the left graph. Scale bars: 50 pA, 20 ms.

## Discussion

The CLA receives dense serotoninergic inputs from the DRN and expresses high levels of 5-HT receptors, including 5-HT1ARs, 5-HT2ARs, and 5-HT2CRs, yet the mechanism by which 5-HT regulates the function of CLA neurons remain ill-defined. Herein, we show that the CLA contains at least four electrophysiologically distinct CLA neurons, RS type I, type II, strong adapting and fast spiking neurons, the excitability of which is differentially modulated by 5-HT. In both RSI and RSII type neurons, 5-HT induces membrane hyperpolarization and depolarization via activation of 5-HT1ARs and 5-HT2ARs, respectively. In contrast, SA neurons are mainly hyperpolarized by 5-HT via activation of 5-HT1ARs. In addition to these postsynaptic modulatory effects, 5-HT redues the strength of glutamate synapses impinging onto all CLA neurons examined. This effect is signaled by activation of 5-HT2ARs. As such, this study begins to decipher complex and multifaceted mechanisms by which 5-HT gates the function of CLA neurons.

The present study reveals that CLA neurons are electrophysiologicaly heterogenous (i.e., RSI, RSII, SA, and FS), which is consistent with earlier electrophysiological studies of mouse CLA neurons (Graf et al., 2020; White and Mathur, 2018). In addition, *post hoc* immunostaining of RSI, RSII, and SA neurons shows that they are large spiny neurons with RSI neurons exhibiting pyramid-like cell bodies, whereas RSII and SA neurons have multipolar and triangular cell bodies, respectively. However, detailed morphological and statistical analysis are required to further confirm these observations. This diverse morphological feature is consistent with earlier studies (Shibuya and Yamamoto, 1998; Rahman and Baizer, 2007; Landzhov et al., 2017) reporting that rat CLA neurons are functionally and morphologically heterogenous group of neurons. Examination of the effects of 5-HT on the resting membrane potential and excitability of CLA neurons reveals that 5-HT exerts cell-specific effects. In RSI and RSII neurons, 5-HT induces either membrane hyperpolarization or depolarization, which indicates that these neurons express both 5-HT1ARs and 5-HT2ARs. Consequently, the overall effects of 5-HT on the resting membrane potential of RSI and RSII, will be dictated by the expression levels of these receptors. The 5-HT induced membrane depolarization and increased excitability will be predominantly observed in neurons expressing higher levels of 5-HT2AR than 5-HT1ARs. In contrast, in SA neurons, 5-HT mainly induces membrane hyperpolarization via activation of 5-HT1ARs, an effect that has been canonically attributed to activation of G-protein gated inward rectifier potassium current (GIRK) (Wong et al., 2024). Thus, in addition to the previously reported inhibitory effect (Wong et al., 2024), the present study shows that 5-HT depolarizes and increases the excitability of RSI and RSII CLA neurons via activation of 5-HT2ARs. Previous studies have shown that RSI and RSII, which are the major outputs of the CLA have distinct projection targets (Erwin et al., 2021) and are involved in different physiological functions. Thus, it will be interesting to decipher the cell-specific effects of 5-HT on the excitability of CLA neurons and their target projections.

The finding that activation of 5-HT2ARs depolarizes RSI and RSII CLA neurons is consistent with the well-established excitatory effect of these receptors in other brain areas, such as the cerebral cortex (Araneda and Andrade, 1991; Béïque et al., 2004; Tang and Trussell, 2015). Indeed, activation of 5-HT2ARs has been shown to depolarize layer V pyramidal neurons of prefrontal cortex, increase their firing activity and induce slow after depolarizing potential (sADP) (Araneda and Andrade, 1991; Andrade, 2011). Such responses are canonical effects induced by other G_q_/G_11_-coupled neurotransmitter receptors, including muscarinic M1 receptors (Haj-Dahmane and Andrade, 1996, 1998). Mechanistically, in other brain areas, activation of G_q_/G_11_-coupled receptors, including 5-HT2ARs induces membrane depolarization and increases neuronal excitability by inhibiting potassium currents (Tanaka and North, 1993; Athilingam et al., 2017) and activating transient receptor potential channels (TRPC) (Zuo et al., 2016). Thus, it is conceivable that a similar ionic mechanism could mediate the excitatory effect of 5-HT2ARs in the CLA and future studies are required to test this notion.

In addition to the postsynaptic effects, we have shown that 5-HT inhibits glutamatergic synaptic drive onto CLA neurons, a finding that is consistent with a previous report (Anderson et al., 2024). The 5-HT induced inhibition of CLA glutamate synapses is mediated by a decrease in glutamate release as indicated by the increase in PPR, CV of eEPSCs and by the decrease in mEPSCs frequency. In addition, the finding that the inhibition of glutamate release is blocked by 5-HT2AR antagonist, MDL 100907 (Sorensen et al., 1993) and mimicked by TCB2, a potent psychedelic 5-HT2AR agonist (McLean et al., 2006), demonstrates that it is signaled by 5-HT2AR. Such conclusion diverges from a recent study suggesting that 5-HT reduces the frequency of sEPSCs via 5-HT2CRs (Anderson et al., 2024). However, it is interesting to note that this inhibitory effect was reported at glutamate synapses of CLA neurons projecting to the anterior cingulate cortex (ACC), which raises the possibility that different 5-HT2R subtypes may exert an input- and cell-specific gating of glutamate synapses in the CLA. However, additional studies are necessary to decipher the precise effects of 5-HT2A/2CRs on the various subgroups of CLA neurons and their afferent inputs.

Previous studies examining the modulation of glutamatergic synaptic transmission by 5-HT2ARs have revealed heterogeneous and sometimes contradictory effects. For instance, in cortical areas, such as the prefrontal cortex, some studies have shown that activation of 5-HT2ARs increases spontaneous excitatory synaptic drive onto pyramidal neurons (Aghajanian and Marek, 1997, 1999; Béïque et al., 2004), by increasing the excitability of local circuits (Béïque et al., 2004). Additionally, it has been shown that activation of these receptors at thalamocortical glutamate synapses of the prefrontal cortex potentiates NMDA-mediated synaptic transmission via an increase in glutamate release (Barre et al., 2016). Whereas results from other studies have shown that 5-HT2ARs induce long-lasting depression of glutamatergic synaptic transmission onto pyramidal neurons via reduced AMPA receptor function (Berthoux et al., 2019). Unlike cortical areas, here, we show that in CLA, 5-HT2ARs reduces the strength of glutamate synapses by inhibiting glutamate release. Interestingly, similar effect has also been reported at glutamate synapses of inferior olive in the cerebellum (Best and Regehr, 2008), where 5-HT2ARs inhibit glutamate release via retrograde eCB signaling. Surprisingly, in the CLA, the present study suggest that the 5-HT2AR-induced inhibition of glutamate release is unlikely to be mediated by PLC-driven retrograde eCB signaling and may involve different downstream signaling pathways. However, additional studies are required to further confirm this concept and elucidate the downstream mechanisms by which presynaptic 5-HT2ARs control glutamate release in the CLA. Collectively, these studies indicate that the multifaceted and often contradictory effects of 5-HT2ARs on the strength of central glutamate synapses in various brain areas most likely reflect differences in their subcellular synaptic distribution and downstream signaling cascades.

The CLA densely projects to various cortical areas (Wang et al., 2017; Atlan et al., 2017) and plays a key role in the regulation of higher-order cognitive functions, including sleep and wakefulness (Narikiyo et al., 2020; Norimoto et al., 2020). Indeed, results from recent studies have shown that the firing activity of CLA neurons projecting to the cortex correlates with cortical slow-wave activity (Marriott et al., 2024). These neurons exhibit higher activity during the synchronized brain state observed in non-rapid eye movement (NREM) sleep and decreased activity during desynchronized brain state in rapid eye movement (REM) sleep (Marriott et al., 2024; Narikiyo et al., 2020). Interestingly, this activity mirrors the firing of DRN 5-HT neurons, which provide major 5-HT inputs to the CLA (Zingg et al., 2018; Peyron et al., 1998), and exhibit lower activity during synchronized brain state (Sakai and Crochet, 2001). The present findings that 5-HT simultaneously inhibits the excitability of CLA neurons and reduce their synaptic strength could constitute a cellular mechanism by which CLA neurons regulate brain states, particularly sleep and wakefulness. Thus, it is tempting to speculate that the low 5-HT tone during NREM (Kato et al., 2022) leads to enhanced activity of CLA neurons, which promote the generation of synchronized brain states (Narikiyo et al., 2020; Marriott et al., 2024). In addition, the 5-HT2ARs-induced gating of excitatory inputs to CLA neurons may also be involved in mediating the hallucinogenic effects of psychedelics. However, future studies are required to elucidate the precise role of 5-HT receptors signaling in the CLA in controlling various brain states.

## Data Availability

The data supporting all the conclusions is found in the manuscript. The raw data is available from the corresponding author upon request.
